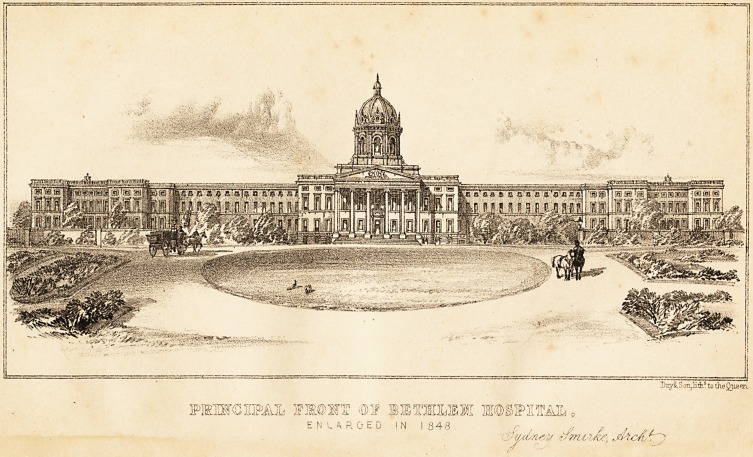# The Great Exhibition of 1851

**Published:** 1851-01-01

**Authors:** 


Day & Son, lith.s to the Queen.

PRINCIPAL FRONT OF BETHLEM HOSPITAL,

ENLARGED IN 1848.

*Sydney Smirke, Arch.t*

### THE JOURNAL

OF

### PSYCHOLOGICAL MEDICINE

AND

### MENTAL PATHOLOGY.

---

JANUARY 1, 1851.

---

### Art. I.—THE GREAT EXHIBITION OF 1851.

Throughout the globe the vestiges of regal dominion are everywhere to be traced upon its decaying surface, nor has Time been able to erase the signs of Art that bestrew the four quarters of the world, and betoken the almost obliterated footsteps of earthly grandeur. In the general calamities of mankind, the death of an individual, however exalted, the loss of an edifice, however famous, are passed over with careless inattention; and we dismiss the deeds of our forefathers with the same indifference as we discard the playthings of our childhood.

Situated to the east of Jordan are the ruins of Djerash, the ancient Gerasa of the Scriptures. They are of transcendent beauty. Their splendour is enhanced by the surrounding solitude in which they repose. Their dignity, however faded and defaced, declares the accomplished taste and intelligence of those by whom they were designed, erected, and possessed. There are triumphant arches, lofty Corinthian columns, long streets, flights of steps, and a row of sixty-seven Ionic columns, arranged in crescentic order; but, with the exception of the Bedouin marauder of the wilderness, shaft, capital, and entablature, upright or prostrate, in confused or broken fragments, remain, unmolested in the sunshine, without a single record to attest their existence on the neglectful page of history.

The Indian architecture, of which no account assures us of its being older or younger than the Egyptian, belongs to the fables of antiquity. The caves of Elephanta and Ellora, and the large pagodas of Mavilipowram, are enigmas to the student of sacred edifices. The natives ascribe their formation to nothing less than the power of supernatural agency. They are unrivalled in their kind; and it is said, that, at first sight, the Tchoultry, or Jun, of Madurah, fascinates the beholder by its magical, scenic, or theatrical air. Longinus, the great critic and unfor-

22048

tunate courtier, a genius that has imparted a perpetual lustre or disgrace to the names of Aurelian and Zenobia, wrote at Tadmor in the desert, whose regal ruins lie, as the poet says, in desolation's sullen majesty. Familiar as we have of late become with Egypt, and all the land of Nile, its sublimity is still its own; and the whirlwind continues now, as it did in the days of Herodotus, and the old time before him, to lash the dust of the sable desert, the same as ever, against the granite of the imperturbable and unaccountable pyramids. Eight cities once flourished at Chiapas and Yucatan, in Central America, of which we know nothing beyond the scanty evidences offered for our speculation by a few scattered mounds, and sites, and fragments, covered with foliage and grass the growth of centuries. Nineveh, long since deceased, but preserved for ages in a state of petrifaction, has just been exhumed, on purpose, as it were, to remind us of Jonah, and render the words of his prophecy as fresh and living as if they had been spoken but yesterday, and were reported, for the first time, among the current news in the columns of to-day's papers.

We talk of the human mind as if it were a permanent thing such as the Peak of Teneriffe or the Polar Star. In common conversation it is mentioned in the most casual way, and rightly so, for we understand each other perfectly well for the time being. By agreement we know the general meaning of the language addressed to us, and assent when it is implied that the mind of a child is not that of an old man, and the understanding of an idiot not identical with that of a person educated and accustomed to the world. But, in the heat of the moment, it escapes our notice, that the mind of one generation is totally different from that of another. Would Cæsar be considered a great man in the present day? Nero or Sardanapalus would probably be hooted down in the streets,—nay, even Napoleon, or Charles XII. of Sweden, would have no opportunity for committing the havoc they did, nor would they any longer be able to leave behind them "a name at which the world grew pale." The mind consists of a series of progressive developments. Suppose a modern drawing-room, with its sumptuous furniture of velvet, silk, glass, gold, china, and rosewood, were to be hermetically sealed up and consigned to the inspection of our descendants some two thousand years yet to come. They would hardly understand its paraphernalia and appointments. It would require time and study to make out the use of this article, and the meaning of that. Their minds would be discordant from ours, and the material substances upon which they employed themselves, or by which they signified their wishes, wants, or desires, would, in process of time, have become so completely new and foreign, that *we* could not understand them, nor *they* us.

The mental constitution of the different ages in the world may be learnt in various ways. The outward signs of architecture is one of them. Like other sciences, it has its alphabet, but the number of its letters is not more than five. Ages, indeed, have been required to invent and exhibit each of them in their primitive and composite forms, beginning with the earliest Egyptian or Coptic, and ending with the model railroad or official residence. The first two letters, called the *horizontal* and *oblique*, constitute the Egyptian and Greek structures. The next two are the circular divided into the *concave*, as the Roman arch, and the *convex*, as the Saracenic, Moorish, and Chinese styles. The fifth is the *vertical*, displayed in those edifices usually denominated Gothic. For the last three hundred years all these modes have been mixed together, and fused into a heterogeneous mass, which, if not without enrichments, conveniences, and beauty of detail, is certainly deficient in greatness of manner, sublimity of conception, and architectonic profiles of light and shade. For the most part, enthusiasm and depth of sentiment have given place to the calls of transient expediency and immediate necessity; and if we examine the Elizabethan or obliquely angular, the Flamboyant or curvilinear, and the modern or rectangular, we must own that this reproach is not without its reason. The railway architecture, the latest of all, is *sui generis*. It may be defined as a combination of the two earliest letters of the architectural alphabet, namely, the oblique and horizontal, described in the form of an inverted cone, truncated, and deeply sunk within the earth, or else, instead of being inverted, thrown upright across spacious vales, aloft in the air like the Roman aqueduct. But chiefly in its size, deportment, and durability, as well as in its elemental configuration, it comes the nearest to the architecture of the pyramids of any structures that have ever yet been raised by man for the last three thousand years. This peculiarity of style argues an intellect of the grandest and most exalted kind. It is rational, geometrical, and correct. Every inch is the result of previous calculation, and the breadth of the base must correspond, not only to the superincumbent weight, but to the velocity of the terrific engines that shall be propelled upon the iron ribs that gird its summit, and tear along the rigid length of its apparently endless causeway. Like the works of the Egyptians, it is colossal; and even the mighty genius of pagan Rome stands midway between the practical genius of England in the nineteenth century and the sublime conceptions of Egypt when the world was young. External shapes are the indications of thought, and the medium by which ideas are conveyed from mind to mind, and from one generation to another. Hence the use of telegraphs, signals, &c. In the mixed architecture of the last three hundred years, we perceive the unsettled mind of Europe subsequent to the great events of the sixteenth century. In the vertical or Gothic that preceded our epoch, we feel, that a religious tone, questionable it may be, but not the less real on that account, pervaded every institution, and professed, in all its actions, chivalrous or not as might be, to aspire to nothing less than to heaven itself. In the Saracenic, Moorish, and Chinese,* we discover the forms of a sensual, if not a feeble race; in the Roman arch, strength of purpose and comprehensiveness of design; in the Greek parallelogram or horizontal square, the most exquisite beauty of outline, described in nothing less than the form of a mathematical problem demonstrated in a pile of marble, polished, sculptured, and ornamented to the last degree of refinement; while in returning to the Egyptian, the type of the railway architecture, we are struck by the immense productions of human ingenuity, intended to be coeval with time, or commensurate with the wide-working operations of Nature herself.

What is proved by architecture, may be likewise gathered from the political and intellectual history of man. They are parallel and consentaneous evidences. When the father of all the faithful, the patriarch Abraham, quitted his native land and kindred, trusting only in divine Providence for support, he travelled with every personal difficulty over distant countries; and when Jacob fled from Esau, he journeyed on foot, and reposed at night on the bare ground, with nothing but a stone for his pillow.†

* Considering the character of the Chinese architecture, which is crescentic, as well as the fashion of their costume, which is loose and flowing, one would infer that the great antiquity of which they boast can scarcely be correct. At all events, the convex architecture dates considerably posterior to the horizontal and oblique, and the Chinese dress is correlative with Persia in the days of Xerxes and Belshazzar, rather than with those of Pharaoh and Nimrod. The Ninevites, Egyptians, and earliest nations were austere and barbaric in their costume, and much more haughty, fierce, and domineering in their physiognomy than the Chinese, as is attested by monumental remains. Lord Macartney, however, fixes the commencement of Chinese history in the dynasty of Chow, 1100 B.C.—a chronology which F. von Schlegel apparently takes for granted. This is contemporaneous with the Judges of Israel, Semiramis, and the Laws of Minos. The Chinese toupet and moustache connects them with the Mongolian and Tartar varieties—it is comic rather than antique. As a psychological phenomenon, burlesque, ridicule, buffoonery, caricature, ribaldry, &c., are significant of debased intellects in individuals, and of declining periods in nations or ages of the world. Athens had passed, or was passing, its climax when Aristophanes wrote, and Terence merely points out the masculine debility of the Romans.

† The story of Abraham, attentively considered, reveals the progress of nations. It is stated, that Abraham, the true successor of Shem, dwelt first at Ur, in Chaldea, thence removed to Charran, in Mesopotamia, and thence again to Sichem, in Palestine. The peaceful lives of the Patriarchs and their longevity are cursorily passed over as their sole and peculiar character; yet they were no strangers to deeper learning, especially to all that relates to sacred traditions and inward contemplation. To them alone are we indebted for the earliest history of the human race. Their opponents, the giants, demigods, or heroes of antiquity, excelled them in science, skill, and energy; it would seem that, at least so far as history has recorded, they were the great masters of nations; and if we examine their physiognomy, as preserved in the ancient monuments of Nineveh and Egypt, they were evidently men of colour, and the descendants of Ham or Cham, which means *burnt* or black.

It required some length of time for the children of Israel to reach the borders of the Red Sea, by marching across the very same desert as that which is now traversed by the overland mail in a few hours.* About five hundred years before Christ, a Phœnician expedition, much to their own surprise, rounded the Cape of Good Hope, by starting from the Red Sea, sailing southward, and coming home along the coast of Africa through the pillars of Hercules; a circumstance which appeared incredible to Herodotus, because they affirmed that at one part of their voyage (*i.e.*, looking westward south of the line) the sun at noonday was on their right hand instead of the left. It is remarkable, that at that time the human mind was making great efforts at advancement. Confucius appeared in China, and Thales and Solon in Greece. It was the dawn of modern philosophy, discovery, and science. The military successes of Alexander the Great, about two hundred years later, are the next to indicate the progress of intelligence; for although the son of Philip of Macedon appears on the stage in the character of a soldier, yet, in the actual result of his arms, we shall perceive that, in spite of his moral derelictions, he was a great statesman, and that the political world experienced a powerful impulse forward in consequence of his comprehensive views, sagacity, and prowess. How he advanced as far as he did with such a vast army—how the commissariat was provided, and the order of march appointed and arranged—are questions for military critics rather than ourselves; but his host was not quite a mob, and if it was less highly organized than similar armaments set on foot at the present day, it bears, notwithstanding, the evident signs of being considerably less barbarous in its movements than were the operations of the allied Greeks before Troy, or the seven heroes of the drama, with their fighting men against Thebes. Before the advent of Christ the Romans

* The eighty-two miles between Cairo (*Musr*) and Suez (*Akabâh*), are run over by a flight of omnibuses, once or twice a month, with the names of Suez and Cairo painted in large letters on their panels. At Stephen's Hotel, in Cairo, there is English crockery, with Sheffield ware, and a London bill of fare—your Arab guide speaks English on the platform at the top of the great Pyramid. The following lines are amusing, and scarcely exaggerated:—

Over the billows and over the brine;
Over the water to Palestine!
Am I awake, or do I dream?
Over the ocean to Syria by steam!
My say is sooth, by this right hand;
A Steamer brave
Is on the wave,
Bound, positively, for the Holy Land!

Godfrey of Bulloigne, and thou,
Richard, lion-hearted king,
Candidly inform us now,
Did you ever—?
No you never
Could have fancied such a thing.

Never such vociferations
Enter'd your imaginations
As the ensuing—

"Ease her," "Stop her!"

"Any gentleman for Joppa?"

"'Mascus, 'Mascus?" "Ticket, please, sir,"

"Tyre or Sidon?" "Stop her," "Ease her!"

"Jerusalem, 'lem, 'lem!" "Shur! Shur!"

"Do you go on to Egypt, sir?"

&c. &c. &c.

had already divided the whole known world, at that time under their sway, into provinces, and intersected the empire in every direction with stone pavements along gigantic causeways, the remains of which are being constantly met with all over Europe. These Roman roads were postal lines, which may be regarded as the germinal idea of a railroad in their solidity, and the electric telegraph in their successful, though imperfect effort at despatch.

Along these ways, the legions with their cohorts of cavalry advanced to the most distant provinces; consuls, prætors, and couriers outstripped the march of the more heavy-bodied troops; and a senatus-consultum was proclaimed in Spain, Gaul, and Byzantium, almost at the same moment of time. Nor did the inroad of the barbarous hordes from the north break up or destroy these sinews of international communication; but only, on the contrary, rendered them useless. For their collective numbers took the place of separate individuals; and the news spread more quickly over the world from mouth to mouth, by private rumour or public report, than it had hitherto done by the means of Government messengers with sealed despatches beneath their cloaks, proceeding post from the Quirinal on the Tiber, or returning thither in haste from headquarters at Antioch, Singidunum, or Colonia Agrippina on the Rhine.* This popular movement was the first idea of a daily newspaper—in one sense it was nothing but gossip, but in another, and a more statesmanlike way of viewing the matter, it was the birth of that intelligence among the masses which constitutes the chief obstacle or assistance—the powerful engine for or against the government of a nation or the world, at the present day. The crusades, indeed, account for them as we may, could never have been suggested, undertaken, or brought to an issue, upon the extensive scale of unanimity by which they were inspired, except for this onward movement of the public mind in the open communication of its ideas; and, in fact, the grand æra had at last arrived when the art of printing was indispensably necessary, not for the erudition, but for the necessities, of mankind—and printing was invented. To this discovery was owing the events, political, literary, philosophical, and religious, of the last four hundred years. The fall of

* When Constantine fled from the snares of Galerius, he left the palace of Nicomedia by night, and travelled post through Bithynia, Thrace, Dacia, Pannonia, Italy, and Gaul, and, amidst the joyful acclamations of the people, reached the port of Boulogne, in the very moment when his father was preparing to embark for Britain. To intercept pursuit, he carried forward with him the post horses all along the line of his journey. His posting proves a highway. With every advantage, it was, however, impossible, that, for the good of future generations, the state policy of the old Roman and Pagan principle should proceed without interruption; for it was nothing else than a grasping, inexorable, selfish cunning. The Barbarians applied the lever that upset this deadening tyranny, and to them we are obliged for our present freedom. The fire still smoulders in the embers of the old pagan world; and slavery of body or mind, wherever it exists, implies that the consuming flame is not yet extinct.

Constantinople, which shook Europe to its centre, might have had some influence in the immediate diffusion of classical learning and the fine arts; but its influence would have been as nothing without the aid of the printing-press. It was the turning point of the intellectual world. The mind must have died out, and perished for the want of its *pabulum vitæ*, the means of diffusing itself in a fixed and definite shape, had it never been discovered. Nevertheless, this discovery, already several centuries old, is only in its infancy—the condensed alphabet of the electric telegraph warns us that a more facile language, and a shorter system of spelling, reading, and writing, than the one we at present employ, awaits the school-days of our more fortunate posterity. Strengthened, at length, by literary knowledge, such as it then was, and instigated by the novel use of the mariner's compass, our ancestors turned their eyes away from home, approached the coasts of Europe, and gazed upon the waves of the Atlantic. Columbus guessed that another continent lay stretched out beyond the utmost verge of the horizon. He guessed the truth. America, the nidus of a nation, and nations of nations, was concealed behind the flowing seas in the direction to which he pointed. Doubted by others, but never doubting himself, thither he sailed, and there he found the land he had anticipated. The scales fell from the eyes of Europe—their vision seemed boundless. Vasco de Gama guessed in another direction with the same unerring ken, and by sailing southward along the west coast of Africa, he rounded the Cape of Good Hope, two thousand years later than the adventurous Phœnicians had already done before him, by sailing in the contrary direction. But the world was as yet but half explored—it remained for our countryman Cook, with a couple of frail ships badly found and worse manned, to circumnavigate the globe in three years, instead of one, as is now done. These discoveries and expeditions changed the mental and physical constitution of man. We are no longer beings of the same species with our forefathers. By means of steam, the hardest mountains oppose no barrier to our progress; the wide Atlantic is reduced to the size of a lake; and the narrow stream of salt-water running between Dover and Calais is nothing more than an extensive esplanade, which may be crossed by any kind of armament, merchandise, or intelligence, at any season of the year, and at any day or hour we please. Proverbs may grow old and be thrown aside, for once it was said, "Time and tide tarry for no man;" but now it is just the reverse, for no man tarries for tide or time.

The growth of mind is extremely slow, gradual, and progressive; and the prominent circumstances which arise in the course of a nation's or an individual's existence, virtuous or vicious as they may be, are not the immediate effects of a sudden impulse, but the deeply-rooted results of ideas inherent and pre-existent in their germs within the very constitution of the person or the community by whom they are eventually displayed. Thus we have traced out the germinal idea—the *punctum saliens*—of newspapers, railroads, the electric telegraph, the discovery of the new world, and the initiative spirit of science—crude and amorphous, indeed, in their first conceptions, but, nevertheless, sufficiently like the future offspring, so as to prevent any mistake in their identity. They are none of them new in themselves—they are five, ten, twenty centuries of age; and if they have not been brought forth into notice in their embryotic or infantile states, it was because, to use a legal phrase, they could not be cognizable as deeds or facts until they had passed into overt acts. The child is not a subject until it has been born, neither is a law, however salutary, efficient until it has been enacted. The philosopher or statesman may foresee events in their causes, but the world at large knows nothing about them until they start into life in a hard, tangible, substantial shape.

Elevated and enlightened in the scale of humanity, the labouring classes, not only in England, but in every other country besides, are feeling their moral ascendancy more perfectly than ever. With the consciousness of their strength is conjoined a sound perception of the benefit of knowledge, sobriety, frugality, and steadiness of deportment. The word liberty, so dangerous in excess, so useful within its proper limits, is being understood and appreciated by them in its most legitimate meaning. They are willing to surrender their freedom to those laws which guarantee the security and exercise of their freedom to them in return. They feel themselves the citizens of a vast community; and they have learnt the dignity of their respective conditions, the importance of peace, and the indomitable force of science and free will united. Even wealth, as wealth, is finding its proper level in society; and one of the most interesting psychological phenomena of the age is, that the fabled riches of Golconda (verified in California) can neither dazzle the senses nor mislead the judgment of the crowd, should they ever be basely employed to purchase their suffrages at the price of their independence. Money, valuable as it is, is nothing more than a commodity in the market to be bought and sold at its marketable value. It is a means to an end.

Were it not for this disposition evinced by the lower orders (as they are somewhat disparagingly termed), which is, in truth, the practical result of centuries spent upon the education of mankind, not only could the present state of society have no existence, but our pen could not describe what in fact did not exist. To ourselves, as psychologists, the past review, such as we have imperfectly rehearsed it, is intensely interesting. Our avocations lead us to meditate on themes like these. We see through the material body, and inspect the mind, the soul, the spirit, within its earthly tabernacle or shrine; and when it agitates itself within its prison of clay, and shines forth in works of art, literature, social improvements, political plans and changes, &c., we admire its human, its divine agency. Never was there an epoch when the rulers and the ruled stood on so equal a footing as the present.

With the present month commences another half-century of the world's progress. Looking back to that which immediately precedes it, we cannot fail to observe the great advances which have taken place in every science calling for the special exercise of the human mind; in the gradual development of moral influences, the decrease of brutalizing amusements, the spread of education and the refinement of our popular recreations, we see a new and wonderful movement in operation; England, headed by her sovereign, engaged in carrying out the proposition of the Prince Consort, erecting a magnificent structure, itself a wonderful triumph of skill, to contain the united riches of the world's intellectual and physical strength; her subjects ever anxious to excel in all that can render her glorious in the arts of peace, striving worthily to compete with foreign and friendly rivals in this pre-eminently ennobling contest; we see every nation and people invited to develop, not only their own well-known industrial resources, but to seek amidst their remote, uncultivated, and almost unknown regions, for some new and useful product of the Creator's infinite wisdom, from which to eliminate some useful material for human industry to display its resources, in order that it may become a source of national and individual wealth.

To the Pyschologist and mental philosopher, this great movement of mankind cannot fail to present many subjects for contemplation. We propose to consider its probable effects upon the mental progress and civilization, and in doing so, we shall first view the matter in its relation to society in the aggregate, and then refer briefly to its influence on individual minds. During the progress of the last fifty years, more than in any other similar period of her history, England has become in an eminent degree a refined and intellectual nation. While the wonders of modern science have engaged her men of genius, educational institutions have so popularized science and the politer arts, that a love of knowledge for its own sake, may be said to be one of the distinguishing characteristics of the age.

The national progress is emphatically benevolent and peaceful; we have seen national and international strife abroad, while our own land has enjoyed an uninterrupted peace; we have seen laws with which intolerance had disgraced our statute-book, gradually expunged; the rigour of the penal laws relaxed, and its extreme penalty repealed for certain offences, which we now regard as trifling in comparison with the punishment formerly inflicted; we have seen modern science engaged in the improvement of our domestic and national comforts; the arts of medicine and surgery rising to perfection; while in no department of philosophy has improvement been so strikingly manifest as in the gradual but steady advance of mental pathology. To it we may look back with pride and satisfaction. We no longer treat our insane fellow-creatures with chains and dungeons; but, regarding them as afflicted fellow-christians, seek, by kindness, to ameliorate their condition, and by skill, to restore them to their wonted places in society.

The Exhibition of 1851 presents itself to us as a practical attempt to gather the fruits of intellectual and industrial progress, to concentrate together the results of the world's industry for the admiration and improvement of the world. The royal personage who proposed it has himself declared it to possess a psychological interest.

"I conceive it," said the Prince, "to be the duty of every educated person closely to watch and study the time in which he lives: and, as far as in him lies, to add his humble mite of individual exertion to further the accomplishment of what he believes Providence to have ordained (*cheers*). Nobody, however, who has paid any attention to the particular features of our present era, will doubt for a moment that we are living at a period of most wonderful transition, which tends rapidly to the accomplishment of that great end to which, indeed, all history points—the realization of the unity of mankind (*great cheering*); not a unity which breaks down the limits, and levels the peculiar characteristics, of the different nations of the earth, but rather a unity the result and product of those very national varieties and antagonistic qualities. The distances which separate the different nations and parts of the globe are gradually vanishing before the achievements of modern invention, and we can traverse them with incredible ease; the languages of all nations are known, and their acquirement placed within the reach of everybody: thought is communicated with the rapidity, and even by the power of lightning. On the other hand, the great principle of division of labour, which may be called the moving power of civilization, is being extended to all branches of science, industry, and art. Whilst formerly the greatest mental energies strove at universal knowledge, and that knowledge was confined to the few, now they are directed to specialities, and in these again even to the minutest points, but the knowledge acquired becomes at once the property of the community at large. Whilst formerly discovery was wrapt in secrecy, the publicity of the present day causes that no sooner is a discovery or invention made, than it is already improved upon and surpassed by competing efforts (*cheers*); the products of all quarters of the globe are placed at our disposal, and we have only to choose which is the best and cheapest for our purposes, and the powers of production are intrusted to the stimulus of competition and capital. So man is approaching a more complete fulfilment of that great and sacred mission which he has to perform in this world. His reason being created after the image of God, he has to use it to discover the laws by which the Almighty governs his creation, and by making these laws his standard of action, to conquer Nature to his use—himself a divine instrument. Science discovers these laws of power, motion, and transformation: Industry applies them to the raw matter, which the earth yields us in abundance, but which becomes valuable only by knowledge: Art teaches us the immutable laws of beauty and symmetry, and gives to our productions forms in accordance with them (*cheers*). Gentlemen, THE EXHIBITION OF 1851 is to give us a true test and a living picture of the point of development at which the whole of mankind has arrived in this great task, and a new starting-point from which all nations will be able to direct their further exertions (*cheers*). I confidently hope that the first impression which the view of this vast collection will produce upon the spectator will be that of deep thankfulness to the Almighty for the blessings which He has bestowed upon us already here below; and the second, the conviction that they can only be realized in proportion to the help which we are prepared to render to each other, therefore, only by peace, love, and ready assistance, not only between individuals, but between the nations of the earth."

To effect this great work, the man of science and the workman, the philanthropist and the speculator, are all united; each class has different objects, feelings, and motives. Whilst the higher class of intellect is employed in the exercise of its most exalted faculty, that of invention, imitative skill is engaged in producing; and this not in our own land alone, but among almost every other nation and people. The effect of this is to call forth in an especial manner the latent talent of many minds, and to develop national and individual energy.

If we would trace the mental progress of a nation, we have two sources of information—viz., its laws, and its popular amusements; the former we shall find appeal more to the animal than to the intellectual man; as soon as the feeling of disgrace appears as a frequent preventive to crime, we have less crime, and punishments become mitigated in severity; and as civilization advances the law becomes less and less severe; industry is protected from lawless riots, and prosperity results. If we would judge of the progress of a nation, we shall find its history recorded in its laws.

The amusements of a people show the intellectual state and condition of the masses; where these are brutalized the shows and popular assemblages partake of that character. In what respect do the bull-fights of Spain differ from the gladiatorial combats of Rome during her decline as a nation? What were the distinguishing characteristics of the dark ages?—crusades, civil wars, and tournaments. What caused Holland, from an almost submerged swamp, to take its place among the commonwealth of nations?—the indomitable energy and industry of its people.

If, then, we regard the Great Exhibition but as a collection of curiosities brought together for the amusement of a popular assemblage, we must regard it with feelings of delight, as pointing out to us the great difference which the advance of civilization has produced upon our national character, in comparison with the more sensual delights of former days.

To see the effect of the proposed Exhibition upon society in the aggregate, it will be necessary for us to view man under two relations; in the barbarous or half-civilized state, on the one hand, and as a member of a civilized and intellectual community on the other; for without thus considering the question we fear our remarks may be misunderstood, and we have no desire to involve it in any doubt, our object being, as before stated, to consider the Exhibition in its purely psychological relations.

There is scarcely a native Indian of North America, or wandering New Zealander, that does not possess some peculiarly simple article of utility, requiring some degree of skill in its formation; and no district of country in any part of the globe exists, which does not afford some useful substance for the use of man. By this phrase we mean, not the mere animal or vegetable which affords an extemporaneous meal to the wandering hunter, but that which might by industry become an article of export to distant lands. One of the great causes of the retardation of these inhabitants of thinly-peopled and savage lands has been, the want of markets for their produce: by creating a demand we increase the supply; by this increase we congregate into a community the hitherto wandering tribes, collected together by the common impulse of natural assistance, and mutual support; civilization and Christian principles become the rule and bond of union, and the wandering race, no longer nomadic and preying upon each other, becomes united in the bond of mutual obligation.

If we contrast wool, as a product of our Australian colonies, with the fishery of the west coast of Ireland, our point will be at once seen to be tenable; the former finds a ready market in this country, while the other, for want of a demand, languishes in obscurity among a peasantry far behind the other inhabitants of the empire in industrial resources.

It is not improbable, that some of these products of natural resources and industrial skill, from localities and sources hitherto unknown, will be brought together at the Exhibition. The present tendency of the English character and enterprise is undoubtedly colonization. We do not enter into the abstract question whether the Anglo-Saxon race is destined to carry civilization into the remote regions of the earth, or not, but we hazard the opinion, that the fruits of the Exhibition will be to transplant from our native land into remote regions the indomitable energy and perseverance of our native industry, and thus we shall open new resources to our colonists; for it must be admitted that these regions have hitherto presented fields of enterprise to the agriculturist rather than to the manufacturer, and that they have tended rather to the depreciation than to the advancement of man's intellectual and scientific resources.

If, on the other hand, we show, that the vast regions and thinly-peopled districts of our colonial empire are productive of the useful raw material, which, instead of wasting its luxuriance in an uncultivated desert, is capable of being formed into articles of utility, we introduce into those colonies the first germ of independent prosperity,—we collect together the industry of its inhabitants into a civilized community, and afford a useful field for the mechanical industry of those who, from having been accustomed to the lighter, but more dextrous manual operations, would take into these distant lands the manufacturing energy of the mother country, a mutual relationship would thus be established, which would materially tend to the improvement of both; our colonies would then be but mere transplantations, if we may be allowed the expression, of portions of our native land; for, by this means, we should establish communities, instead of sending out wandering emigrants, who, from want of civilized society, necessarily relapse from the high-toned morality so conspicuous in the country of their forefathers, into a refined state of barbarism.

It may be objected that the vices of society, and the less refined pleasures of popular assemblages, would accompany them, but these are never so potent, as when civilized man finds himself located among the barbarous tribes of a half-civilized community, living apart from his brethren in a log hut, and without rational means of enjoyment. His daily agricultural operations produce monotony, listlessness, and enervation, and instead of being the intellectual head, and spiritual pride of the rude tribes surrounding him, he not unfrequently refines upon their barbarism, and brings discredit not only upon himself, but upon the profession of religion which he disgraces by his vices, and the utter neglect of everything sacred. Missionary enterprise is productive of vast good. Until the barbarism of civilization intrudes nomadic colonization into the vast regions of thinly-peopled districts, the solitudes of the interior of the colony teem with wandering flocks, attended each by its solitary herdsman; the fruits of the earth, and the flowers of the field, exist there in unknown luxuriance and inutility; its rivers are not navigated, its mineral riches lie undiscovered, its people wander about with the freedom of the early inhabitants of the world, without their pastoral simplicity; visited by occasional trading-vessels, they imbibe all the vices, without the virtues, of a civilized community, and are known but little in the parent state; but by their export of the bounteous gifts of Providence, in the rough and unmanufactured state, they progress in wealth and importance, but with the exception of a few maritime towns, their population is scattered through an extensive district of country, there is no community of sentiment, no bond of union, no manufactories to form the nuclei of civilization : each new settler proceeds still further into the land than the one who preceded him, anxious only to secure a large and unsettled tract of country upon which to feed his sheep without hindrance from his neighbours.

To the eye of the psychologist this system presents many salient points of attack. If the great duty of civilized man is to increase in knowledge and usefulness, to improve his mental faculties, by cultivating the higher attributes of his reasoning powers, and to associate into communities for mutual benefit and support, surely any means which promises an amendment will gain the commendation and support of all well-wishers to mental improvement and civilization.

If we were asked what we conceived to be one of the great advantages likely to result from the Exhibition of Industry, we should place in a prominent point of view the good effect likely to result to our colonial empire. The wealth of our colonies has been hitherto in a great degree undeveloped, their more natural productions forming the greatest amount of their exports. We regard the Exhibition as eminently calculated to develop their manufacturing capabilities. That it will lead to a new class of emigrants, whose objects will be more immediately directed to a higher description of manufactures, we confidently believe. We think this will be the result of the Great Exhibition.

We feel that the effects of the Exhibition upon the barbarous or half-civilized communities will be to develop industry and natural resources; in other words, to open new fields of profitable enterprise and labour. These will be subservient to the improvement of the intellectual and moral faculties of the people generally, will introduce the blessings of civilization, and tend to the expansion and elevation of the human mind. England will feel that she was mainly instrumental in showing to the world, that true happiness consists in peaceful labour, producing contentment as far as the mere animal appetites are concerned, but leaving still the thirst for intellectual and moral culture inseparable from a life of active energy.

Intellectual advancement is the natural result of industry. Man feels, as he progresses in the latter, an innate desire for the advantages of the former. It is the part of human nature constantly to progress: industry leads to association, and emulation directs individual and national progress. Once direct the energy of man to the accomplishment of a given task, let him clearly understand the *means* by which it may be effected, and he brings to bear upon it his reasoning faculties, and however untutored he may be, however unaccustomed to this mental exercise, he will endeavour to perform the work, and take a pride in its fulfilment. Thus pride, another attribute of human nature, is brought to bear upon an industrial operation, and converted from an uncivilized appetite into a domestic virtue.

Having thus briefly reviewed its effects upon a state of society removed in a great degree from the great centres of civilization, and as it were struggling between barbarism and civilization, our next point will be the consideration of the effects of the Great Exhibition upon society at large, using this term, not in an exclusive application to our own country, but to the civilized nations of the world.

Emulation is one of the strongest passions of the human mind, giving rise in the barbarous state of a nation to personal courage and bravery, and in the more civilized, to industry and mental advancement. Let us not be misunderstood by placing bravery and personal courage in opposition to industry and mental advancement. We wish merely to contrast the virtues of two distinct phases of society, the educated and uneducated, the warlike and the peaceful: by making bravery the chief personal virtue the warlike nation progresses in its conquests, the higher attributes of civilization meet with no reward; while, on the other hand, an industrious people, by the exercise of the mental faculties upon more useful employments, retain all the courage of the more warlike, pursue an uninterrupted course of national prosperity, constantly advance in intellectual and moral supremacy, and when roused to defend their country from foreign aggression, dignify war, by bringing to bear upon its horrors the more ennobling characteristics of humanity.

The Great Exhibition is only an attempt to inculcate this lesson —the industrial energy of the people is the great safeguard of a nation, its protector against intestine commotions, its security from foreign aggression; and our native land, in thus collecting together the products of the world's industry in her own metropolis, will be enabled to show, not only the results arrived at, through the medium of the Prince Consort's intellectual conception, but also the fruition of many ages of internal peace and national prosperity.

We may be permitted here to draw a contrast between England in the reign of Victoria, and Rome in the Augustan age, when both nations, in the enjoyment of a universal peace, were preparing to collect together in their capitals the surrounding nations and people, for the gratification of their senses; the inhabitants of the imperial city brings into the arena the wild beast of a conquered territory, to fight with the image of its Creator; debasing the minds of the spectators by the exhibition of the brutalizing combat, and writing in words of blood, that physical force alone was the passport to fame. On the other hand, the great Queen of a free and unshackled people collects together the nations to display the arts of civilization, the riches not only of the material world, but of man's cultivated intellect; teaching them that the soul, the bright and ennobling attribute of man, presides over and directs the popular amusements of a Christian civilization.

Contrast the Colosseum, still great in its ruins, vast in its magnificence as the nation which raised it, with the crystal palace. Both are fitting types of the civilization they represent. The one rearing its gigantic arches amidst the groans of the slaves brought from their native wilds to grace the triumphal progress of their imperial conqueror, and then compelled to toil, that they might rear the monument of their conqueror's greatness; the other, like Solomon's temple, the type of wisdom and a divinely inspired civilization. In that cloud-capped wilderness of human toil what a contrast, or rather series of contrasts, will here be presented; the riches of the world's industry in their varied relation to genius, energy, and skill, the productions of every nation, mineral, vegetable, and animal, will be displayed beneath that glass-clad roof.

If we pursue our inquiry, we shall find that the preparations for the Exhibition have had a high value in reference to the mental progress of our people; it has brought the prince and the peasant, the peer and the merchant, the man of genius and the operative mechanic, into a closer bond of union: establishing, as it were, a great mental republic—a republic unsullied by political animosities, but dignified by the common cause of intellectual and moral advancement, which teaches men that true loyalty is fostered and encouraged by the industry of the people, and that for the first time in the history of mankind the Lady Sovereign of a free, united, and intelligent people will dispense the rewards of industry; will preside as the supreme head of the industrial republic of the universe. Ages and generations of people may pass away and be forgotten, our very nation may in its turn be overcome with decay, and, like modern Greece, its inhabitants may point out the sites of its former cities, but the enduring traditions of 1851 will still remain, the medals then distributed will perhaps embellish the cabinet of the collector in a nation now unknown, whose future cities are but log huts—whose inhabitants, wandering tribes; its palace of industry will vanish with the occasion that called it into existence, but the effects will remain; the example then set will have an influence upon the succeeding people and nations, and redound to the honour and happiness of the world at large.

The formation of local committees, not only in this country but abroad, has given an individual spirit to the collective exertions,—has developed more strongly the industrial energies of the working classes, and has, moreover, thrown into the work a co-operation which would ve been sought in vain without such a system; and, as springing from this co-operation, the formation of clubs and associations, having for their principal objects the cheap transit of the community and their support while in London, are specially interesting as indicating the mental progress of the people.

In conclusion, we may observe that, the "Crystal Palace" is an outstanding sign of the mind of the age. It could not have taken place half a generation back. It was impossible three hundred years ago. It could not have been dreamt of by the fondest enthusiasts, nor imagined by the chivalry of the middle ages. The Goths were creatures of flesh and blood, without ideas; and Marcus Aurelius, and Antoninus Pius could have formed no notion of it, nor have executed their notion, had they formed it. Pliny was a naturalist, and Seneca a moral philosopher; but neither moral philosophy nor natural science had reached so far as this. Trajan might build a column at Rome, or throw a bridge across the Danube,—Hadrian might plan, build, and furnish a villa for his own particular use and gratification;—but to collect the industry of all nations within the capacious area of a single vestibule, was a feat beyond their power.

Even the design of the edifice is indicative of the national mind. Beneath its shadow, or rather within its lantern-skylight, will be brought together persons from every quarter of the world; and specimens of every work of art from every nation upon the face of the earth will be exhibited, in order that they may be surveyed and examined at leisure by the eye of every native by whom they have been severally contributed. The ultimate result must be as extensive as the design. It is an event that cannot be overlooked. The difference between man and man consists, not in the difference of costume, language, clime, and nation, but in the different advances effected by each in the progress of civilization, commerce, and the mutual, the invaluable bonds of good-will and peace.

To every epoch there is annexeda particular character, tone, or temper of mind, usually denominated the spirit of the age, and used for the time being, as the standard by which everything else is to be judged of in the locality, nation, or empire where it reigns. In the post-diluvian period, the spirit of the age was that of national aggrandizement and earthly glory (*mundus et brevis gloria ejus*), against which stood out in contrast, the simple, rural, individual lives of the patriarchs. Once was offered to one, the kingdoms of the world, and all their glory, on the revolting condition of falling down and worshipping their detestable lord and master; but stupendous were the moral results. Rome was at its zenith when the spirit of the age was, for the first time in history, rebutted by the spirit of forbearance. In that critical moment, on the top of a lofty mountain, apart from the seat of government, the destiny of man was changed for ever. Two powers were generated in conflict that arose and overthrew the pagan world—the one emerged from the catacombs beneath the foundations of the Eternal City, and the other rushed down and stormed the fair plains of the south, from the cimmerian darkness of the north. The spirit of the age became mixed, and produced the spirit of theology, the spirit of reformation, the spirit of literature, and the spirit of war. The spirit of the present age is that of science, exact, inquisitive, and severe; and should it give rise to the spirit of peace among the nations of the earth, and they should agree to melt their cannon into ploughshares, and turn their swords into sickles, it would be a marvel, not beyond the words of prophecy, indeed, but apparently not as yet within the range of possibility. But the spirit of poverty must precede that of peace, for the thirst of gold is the cause of war, and covetousness lies at the root of every contest. If ever the kingdoms of the world, and all their glory, shall adopt the precepts and counsels of the Gospel as the basis of their government, then would the spirit of the age become the spirit of eternity—a vision which the too palpable imperfections of our nature forbid us from contemplating.

## Figures and Tables

**Figure f1:**